# Comparing Cathelicidin Susceptibility of the Meningitis Pathogens *Streptococcus suis* and *Escherichia coli* in Culture Medium in Contrast to Porcine or Human Cerebrospinal Fluid

**DOI:** 10.3389/fmicb.2019.02911

**Published:** 2020-01-14

**Authors:** Marita Meurer, Nicole de Buhr, Linn Meret Unger, Marta C. Bonilla, Jana Seele, Roland Nau, Christoph G. Baums, Thomas Gutsmann, Stefan Schwarz, Maren von Köckritz-Blickwede

**Affiliations:** ^1^Department of Physiological Chemistry, University of Veterinary Medicine Hannover, Foundation, Hanover, Germany; ^2^Research Center for Emerging Infections and Zoonoses (RIZ), University of Veterinary Medicine Hannover, Foundation, Hanover, Germany; ^3^Department of Neuropathology, University Medical Center Göttingen, Georg-August-University Göttingen, Göttingen, Germany; ^4^Department of Geriatrics, Evangelisches Krankenhaus Göttingen-Weende, Göttingen, Germany; ^5^Institute of Bacteriology and Mycology, Center for Infectious Diseases, Faculty of Veterinary Medicine, University of Leipzig, Leipzig, Germany; ^6^Research Group Biophysics, Research Center Borstel, Borstel, Germany; ^7^Department of Veterinary Medicine, Institute of Microbiology and Epizootics, Center for Infection Medicine, Freie Universität Berlin, Berlin, Germany

**Keywords:** MIC, AMP, LL-37, PR-39, *Streptococcus suis*, *Escherichia coli*, cerebrospinal fluid (CSF)

## Abstract

Host defense peptides or antimicrobial peptides (AMPs), e.g., cathelicidins, have recently been discussed as a potential new treatment option against bacterial infections. To test the efficacy of AMPs, standardized methods that closely mimic the physiological conditions at the site of infection are still needed. The aim of our study was to test the meningitis-causing bacteria *Streptococcus suis* and *Escherichia coli* for their susceptibility to cathelicidins in culture medium versus cerebrospinal fluid (CSF). Susceptibility testing was performed in analogy to the broth microdilution method described by the Clinical and Laboratory Standard Institute (CLSI) to determine minimum inhibitory concentrations (MICs) of antimicrobial agents. MICs were determined using cation-adjusted Mueller–Hinton broth (CA-MHB), lysogeny broth (LB), Roswell Park Memorial Institute medium (RPMI) or Dulbecco’s Modified Eagle’s Medium (DMEM) (the latter two supplemented with 5% CA-MHB or blood) and compared with MICs obtained in porcine or human CSF. Our data showed that MICs obtained in CA-MHB as recommended by CLSI do not reflect the MICs obtained in the physiological body fluid CSF. However, the MICs of clinical isolates of *S. suis* tested in RPMI medium supplemented with CA-MHB, were similar to those of the same strains tested in CSF. In contrast, the MICs in the human CSF for the tested *E. coli* K1 strain were higher compared to the RPMI medium and showed even higher values than in CA-MHB. This highlights the need for susceptibility testing of AMPs in a medium that closely mimics the clinically relevant conditions.

## Introduction

The increase in antimicrobial resistance is an emerging problem and requires new strategies to combat bacterial infections (Kim, 2013). Fighting infections with the application of antimicrobial peptides (AMPs) is considered a promising new strategy. As AMPs have antimicrobial and immunomodulatory properties, they are of interest as novel therapies or for supplementing existing treatments. Isolation or synthetic production of natural AMPs and designing new optimized AMPs are already possible and affordable (Wang et al., 2016).

An important group of AMPs are the cathelicidins. They consist of 12–100 amino acids and occur in all vertebrate species (reviewed by Tomasinsig and Zanetti, 2005). Their positive charge allows them to bind to the negatively charged bacterial membranes and subsequently lyse them. Thus, cathelicidins can exhibit an immediate antimicrobial effect. In addition, cathelicidins have been shown to modulate the innate immune response against infections (reviewed by van Harten et al., 2018). Current research is focusing on the synthetic generation of smaller peptidic analogs of cathelicidins with improved antimicrobial activities as useful tools for evaluating cathelicidins in the treatment of severe infections (reviewed by Wang et al., 2019).

The only human cathelicidin LL-37 and the porcine cathelicidin PR-39 are produced among other cells in neutrophil granulocytes (reviewed by Zanetti et al., 1995). Neutrophil granulocytes can penetrate into the cerebrospinal fluid (CSF) via the blood-CSF barrier (Wewer et al., 2011), where they release the cathelicidins to fight the infection (de Buhr et al., 2017). Therefore, cathelicidins are of special interest in bacterial infections of physiological niches in which many antibiotics poorly penetrate, such as the CSF (Nau et al., 2010; van Harten et al., 2018). However, cathelicidin activity against meningitis-causing pathogens in the CSF is poorly understood.

The present study focuses on two different meningitis-causing pathogens, the Gram-positive *Streptococcus suis* and the Gram-negative *Escherichia coli*. *S. suis* causes major economic losses in the swine industry. The pathogen can also affect humans, especially those who live or work in close contact to pigs, such as farmers, slaughterhouse staff and veterinarians. *S. suis* outbreaks in humans are mainly reported from Asian countries (Wertheim et al., 2009; Feng et al., 2014; Rajahram et al., 2017), but single infections are also reported in other regions (Goyette-Desjardins et al., 2014; Eisenberg et al., 2015). Serotype 2 is most common in both humans (74.7%) and pigs (27.9%) worldwide. However, in particular in pigs, the serotype 7 is leading to increasing economic problems (6.7% in Europe, Goyette-Desjardins et al., 2014).

Another human meningitis pathogen mainly in newborns and in elderly persons is *E. coli* K1, which is responsible for many severe cases of neonatal sepsis and meningitis (Ku et al., 2015). The susceptibility of this pathogen to human cathelicidin LL-37 is unknown so far. The avirulent and non-meningitic strain *E. coli* K12 is known to be susceptible to cathelicidins (Duplantier and van Hoek, 2013; Veldhuizen et al., 2014).

In order to search for new therapeutic candidates and to test the efficacy of AMPs, standardized methods closely mimicking the physiological conditions at the site of infection are required. The Clinical and Laboratory Standard Institute (CLSI) recommendations for antimicrobial susceptibility testing (CLSI, 2018) are followed in diagnostic laboratories for classical antimicrobials worldwide. However, it is important to mention that some antimicrobial agents possess antibacterial activity *in vivo*, have a therapeutic effect, even when the disease-causing bacteria are tested as being resistant against the infectious agent *in vitro*, since they act differently in the physiological environment and in broth (Ersoy et al., 2017). To determine the minimum inhibitory concentration (MIC), usually cation-adjusted Mueller–Hinton broth (CA-MHB) is used. This medium is optimized for bacterial growth and not for the tissue conditions under which antimicrobial agents act. Therefore, Kumaraswamy et al. (2016) tested the susceptibility of *Stenotrophomonas* in Roswell Park Memorial Institute medium (RPMI) enriched with 10% lysogeny broth (LB) and found significantly lower MICs than in CA-MHB.

In this study, we determined MICs of meningitis pathogens for LL-37 and PR-39 using CA-MHB, LB, RPMI or Dulbecco’s Modified Eagle’s Medium (DMEM) media (the latter two supplemented with 5% CA-MHB or blood) and compared them with MIC values obtained in porcine or human CSF, too.

## Materials and Methods

### Bacterial Isolates

Five different bacterial strains were used: two different *E. coli* strains, two *S. suis* strains and *Staphylococcus aureus* Newman Δ*dlt* (dlt operon is encoding proteins mediating D-alanylation of wall teichoic acids). The strains originated from the following sources:

***Escherichia coli* K12** (serotype O rough:H48) typified by the National Reference Laboratory for *Escherichia coli* of the German Federal Institute for Risk Assessment (BfR). The avirulent and non-meningitic strain *E. coli* K12 is known to be susceptible to cathelicidins and served as a control (Duplantier and van Hoek, 2013; Veldhuizen et al., 2014).

***Escherichia coli* K1** (serotype O18:K1:H7) was originally isolated from the CSF of a child with neonatal meningitis [gift from Dr. Gregor Zysk, Institute of Medical Microbiology, Düsseldorf, Germany (Ribes et al., 2013)].

***Streptococcus suis* serotype 2 strain 10** is an *mrp*^+^, *epf*^+^, *sly*^+^ strain of multilocus sequence type (ST) 1. It was kindly provided by Hilde Smith, DLO-Lelystad and had been isolated from a pneumonia case in a pig (Vecht et al., 1996; Smith et al., 1999). Furthermore, this strain has been shown to be highly virulent in experimental infections of piglets leading to meningitis (Baums et al., 2006; Silva et al., 2006).

***Streptococcus suis* serotype 7**, published as strain 13-00283-02, is a *mrp*^+^ strain of ST 29. It was isolated from the brain of a pig with meningitis in 2013 in Germany (Rieckmann et al., 2018).

***Staphylococcus aureus* Newman Δ*dlt*** (Peschel et al., 1999) was used as a reference strain for LL-37 (Blodkamp et al., 2016).

All strains were grown from frozen glycerol stocks on blood agar plates (Columbia Agar with 7% Sheep Blood; Thermo Scientific^*rmTM*^ PB5008A) for 16 to 20 h at 37°C.

### Antimicrobial Peptides

Two different cathelicidins were used, the human cathelicidin LL-37 (LLGDFFRKSKEKIGKEFKRIVQRIKDFLRNLVPRTES) and the porcine cathelicidin PR-39 (RRRPRPPYLPRPRPP PFFPPRLPPRIPPGFPPRFPPRFP). The synthesis of peptides is described in Supplementary Material and Methods.

### Media

CA-MHB (Oxoid CM0405, MgCl_2_.6H_2_O Sigma Aldrich M2670, CaCl_2_.2H_2_O Merck 2382) was used as the standard medium as it is recommended by the CLSI. Mueller-Hinton broth (MHB) (Oxoid CM0405) without cation adjustment was used to test the reference strain *S. aureus* Newman Δ*dlt*.

RPMI (Gibco, 11835063) and DMEM (Gibco, 31053028) were used, supplemented with 2.5, 5, 7.5, 10, 15 and 20% CA-MHB or 5% laked horse blood (Oxoid, SR0048C).

Lysogeny broth [10 g Tryptone (Roth 8952.2), 5 g yeast extract (Roth 2363.2), 5 g NaCl (Roth HN00.3) in 1 L deionized water] was used to generate growth curves and MIC values.

### CSF Samples

The anonymized leftovers of CSF samples of patients receiving diagnostic lumbar punctures were pooled and utilized for the MIC determination. The patients did not suffer from any infectious disease and had no CSF pleocytosis. The non-commercial use of pooled CSF leftovers was approved by the Ethics Committee of the University Medical Center Göttingen, Georg-August-University Göttingen, Germany.

Porcine CSF was taken from non-infected control animals of an animal experiment registered at the Lower Saxony State Office for Consumer Protection and Food Safety (LAVES) (Niedersächsisches Landesamt für Verbraucherschutz und Lebensmittelsicherheit) under no. 33.12-42502-04-12/0991.

Human and porcine CSF were stored at −80°C.

### Susceptibility Testing

Minimum inhibitory concentration values were determined via broth microdilution as described in the document VET01A4 of the Clinical and Laboratory Standards Institute (CLSI, 2018). The method was adjusted in order to test different media and CO_2_ contents. AMP susceptibility testing was repeated for each different approach three times on independent occasions.

The strains were plated on blood agar plates (Columbia Agar with 7% Sheep Blood; Thermo Scientific^TM^ PB5008A) and incubated at 37°C overnight. Colony material was suspended in 0.9% NaCl-solution to an OD_625nm_ of 0.08–0.13 corresponding to 1 × 10^8^ CFU/mL. These suspensions were also used to assess the CFU/mL after sequential ten-fold dilutions in PBS to 10^–5^ and 10^–6^ and plating these dilutions on blood or LB agar plates [10 g Tryptone (Roth 8952.2), 5 g yeast extract (Roth 2363.2), 10 g NaCl (Roth HN00.3) 15 g Agar (Roth 2266.3) in 1 L water] for counting the colonies.

The bacterial suspension was diluted in the different test media by 1:100. Microtiter plates (Greiner bio-one, PS, U-bottom 650101, without lid) were prepared with 50 μL test medium and AMPs in a two-fold dilution series. The concentrations of the AMPs per well ranged from 0.06 μg/mL to 128 μg/mL for LL-37 and up to 256 μg/mL for PR-39. Then, the prepared microtiter plates were inoculated with 50 μL bacterial suspension to yield a final concentration of the bacteria of 5 × 10^5^ CFU/mL in a total volume of 100 μL. After covering the plates with foil, these were then incubated with or without 5% CO_2_ at 35°C for 20 h. The MIC was determined as the lowest AMP concentration which yielded no visible bacterial growth. Furthermore, the OD_620nm_ was determined with a Tecan Plate Reader (Infinite 200 Pro). Growth controls (without peptide) and medium controls (without bacteria) were included.

### Statistical Analysis

The statistical analysis was performed using the Kruskal–Wallis test with the Dunn’s comparisons test being subsequently used for MIC testing in different media and atmospheres. The MIC values were analyzed with GraphPad Prism Version 8.0.1.

## Results and Discussion

### Bacterial Growth in Different Media

The aim of our study was to test the MICs of LL-37 and PR-39 with broth microdilution in accordance with the CLSI recommendations (CLSI, 2018) in CA-MHB and compared with LB, various well-known cell culture media and human or porcine CSF. We selected the media based on the recommendation of the CLSI. However, CA-MHB alone does not correspond to body fluids in any way. Therefore, we wanted to ascertain whether differences in the MIC values occur when media that resemble more closely the physiological conditions at the site of infection are used. Since the strains only showed a very low optical growth in cell culture media (Supplementary Figures S1A–D), we enriched the cell culture media with CA-MHB. RPMI and DMEM are the most frequently used cell culture media when working with eukaryotic cells to mimic their physiological environment. Their composition is closer to the physiological situation than broth made from dehydrated beef, casein and starch. LB is a broth often used for cultivating different bacteria in the laboratory. In addition, its composition does not correspond to that of body fluids but guarantees good bacterial growth (Supplementary Figures S1E–L).

How well cell culture media reflect the physiological conditions is a highly discussed topic (McKee and Komarova, 2017). In normal CSF, the Ca^2+^ concentration is approximate half of the serum concentration, i.e., corresponds to the fraction unbound in serum. The Mg^2+^ concentrations in normal CSF are slightly higher than the corresponding serum levels. When the blood CSF barrier is damaged as for example during active tuberculous meningitis, the Ca^2+^ concentrations only slightly rise and the Mg^2+^ concentrations only slightly decrease compared to the normal serum concentrations in contrast to the increasing protein content in the CSF of patients (Hunter and Smith, 1960). Thus, RPMI reflects the low ion composition of human CSF better than DMEM. By adding CA-MHB to RPMI, the low content of Ca^2+^- and Mg^2+^-ions is also brought closer to the values detectable in CSF of patients (Hunter and Smith, 1960). We also tested supplementation with 5% lysed horse blood for *S. suis* as recommended by the CLSI for streptococci. Here, we detected efficient growth, even higher OD with 5% lysed horse blood after 20 h than with CA-MHB or RPMI medium supplemented with 20% CA-MHB (Supplementary Figures S1E–H). To show the number of bacteria in different broths, we determined the CFU/mL after a 6 h and 22 h incubation period. Therefore, we plated in serial dilution on Columbia blood agar plates and counted the CFU/mL to show a relation between OD and CFU/mL (Supplementary Table S2). Finally, we confirmed efficient growth in the used media for subsequent MIC testing.

### MIC Testing of *S. suis* and *E. coli* in Different Media

**FIGURE 1:**
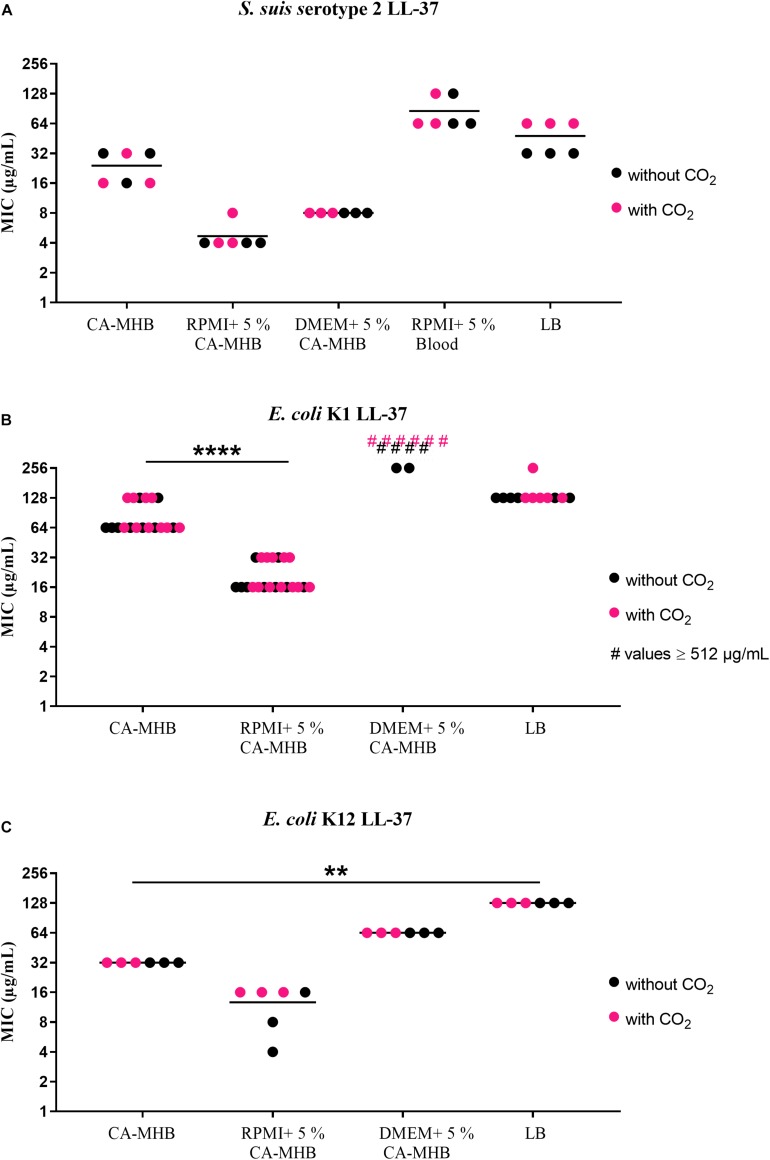
MIC testing with LL-37 in accordance with CLSI recommendations by broth microdilution in different media (data shown are values from three independent experiments). **(A)** *S. suis* serotype 2, **(B)** *E. coli* K1, and **(C)** *E. coli* K12 against LL-37. Statistics: Kruskal–Wallis test with subsequent Dunn’s comparisons test ^∗∗^*p* < 0.0021, ^∗∗∗∗^*p* < 0.0001.

**FIGURE 2:**
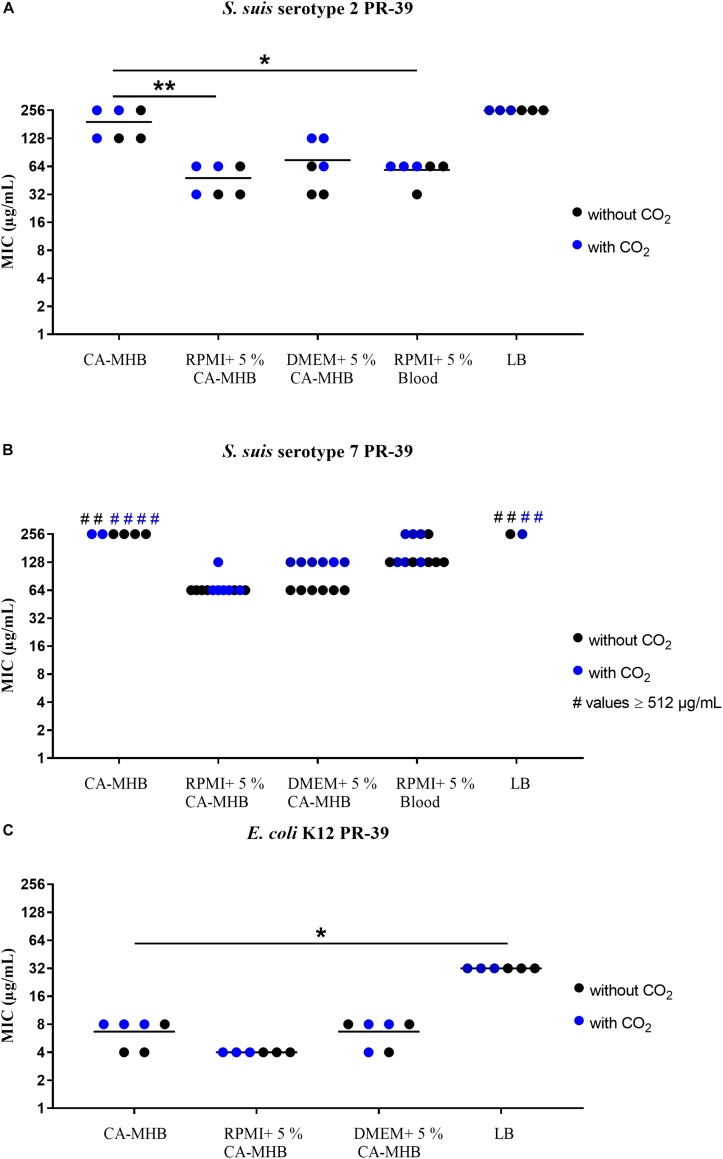
MIC testing with PR-39 in accordance with CLSI recommendations by broth microdilution in different media (data shown are values from three independent experiments). **(A)** *S. suis* serotype 2, **(B)** *S. suis* serotype 7, and **(C)** *E. coli* K12 against PR-39. Statistics: Kruskal–Wallis test with subsequent Dunn’s comparisons test ^∗^*p* < 0.0332, ^∗∗^*p* < 0.0021.

The results of MIC testing in different media are displayed in Figure 1 (LL-37) and Figure 2 (PR-39). For LL-37, *S. suis* serotype 2 (Figure 1A) showed MICs of 16 or 32 μg/mL in CA-MHB. In RPMI + 5% CA-MHB (RPMI + CA-MHB) or DMEM + 5% CA-MHB (DMEM + CA-MHB), the MIC was lower (4 or 8 μg/mL). However, in RPMI medium with 5% lysed horse blood, the MIC was higher at 64 or 128 μg/mL. For *E. coli* K1 (Figure 1B), the MIC in CA-MHB revealed values of 64 or 128 μg/mL; for DMEM + CA-MHB, even higher values were measured; for RPMI + CA-MHB an MIC of 16 or 32 μg/mL was detected. The MIC for *E. coli* K12 (Figure 1C) was 32 μg/mL in CA-MHB and between 4 μg/mL and 16 μg/mL in RPMI + CA-MHB, whereas the MIC in DMEM + CA-MHB was 64 μg/mL.

Very high MICs were found when testing *S. suis* serotypes 2 and 7 against PR-39 in CA-MHB and LB medium (Figures 2A,B). In CA-MHB, MICs of 128 or 256 μg/mL were seen for *S. suis* serotype 2 (Figure 2A); in RPMI + CA-MHB and RPMI supplemented with blood the MICs were only 32 or 64 μg/mL. For DMEM + CA-MHB an MIC of 128 μg/mL was also obtained. *S. suis* serotype 7 (Figure 2B) also revealed high MIC values of 256 or ≥512 μg/mL in CA-MHB as well as in LB. In RPMI + CA-MHB and DMEM + CA-MHB, values of 64 or 128 μg/mL were observed. In RPMI supplemented with blood, MICs of 128 or 256 μg/mL were seen. *E. coli* K12 (Figure 2C) showed lower values of 4 μg/mL and 8 μg/mL with PR-39 and almost equal values for all media and incubation methods. In LB, higher values of 32 μg/mL were obtained for PR-39. *S. aureus* Newman Δ*dlt* served as the control strain and showed expected low MIC values of between 4 and 16 μg/mL (Supplementary Table S1).

In summary, relatively high MICs were found for the *S. suis* serotype 2 and the *E. coli* strains to LL-37 in CA-MHB, as well as for both *S. suis* serotypes to PR-39 (Figures 1, 2). Only the *E. coli* K12 strain tested with PR-39 showed a lower MIC in CA-MHB. For *S. suis*, it may be speculated as to whether the cysteine protease ApdS is highly expressed in the serotype 2 strain in the respective media with high MIC values and whether it destroys LL-37 (Xie et al., 2019) thereby leading to higher MIC values of the bacteria.

### MIC Testing in the Presence or Absence of 5% CO_2_ Incubation

Before testing the MICs in CSF, we detected that there were no significant differences between tests with or without 5% CO_2_ incubation for all strains and media tested (Figures 1, 2). This is important to know since the ion and protein concentration in CSF is significantly lower than in blood leading to a severely limited buffer capacity. If CSF is removed from the body and exposed to the atmosphere, CO_2_ diffuses from the liquid and the pH value rises to an unphysiological value. This process is reversed by incubation in a CO_2_-enriched atmosphere (Cunniffe et al., 1996). Therefore, all MIC testing with CSF were performed in a CO_2_-enriched atmosphere.

### MIC Testing in Porcine and Human CSF

Finally, we performed similar assays as described above in human and porcine CSF to test the MIC in a physiological medium. To prove that there was not a high amount of LL-37 in the pooled human CSF used for the assay, a dot-blot analysis using mouse anti LL-37 antibody was performed (Supplementary Material and Methods). Our result confirms that the concentration of LL-37 is less than 20 μg/mL in uninfected human CSF (Supplementary Figure S2). This result goes in line with low amounts of LL-37 in non-infected CSF (Brandenburg et al., 2008). For MIC and growth curve experiments, CSF of seven pigs was pooled. The PR-39 ELISA (see Supplementary Material and Methods) showed a PR-39 concentration below the detection limit of 0.078 ng/mL for six of these animals. One pig showed concentration of 0.246 ng/mL PR-39 in CSF. These data indicate that no relevant concentrations of the peptides were in the CSF used for the following MIC assays.

For investigating the human peptide LL-37, we used human CSF and for investigating the porcine peptide PR-39, we used porcine CSF. Since we had only a limited amount of CSF available, we tested *S. suis* serotype 7, which does not play a role in human meningitis (Goyette-Desjardins et al., 2014), only in porcine CSF and against the porcine cathelicidin PR-39 and the human *E. coli* K1 in human CSF and against the human cathelicidin LL-37.

As control experiment, growth curves of the meningitis-causing strains in human or porcine CSF were performed (Supplementary Figures S1M,N). Although the growth in CSF was not similar to those in broth or enriched cell culture media, growth was efficient to enable effective MIC testing in CSF.

**FIGURE 3:**
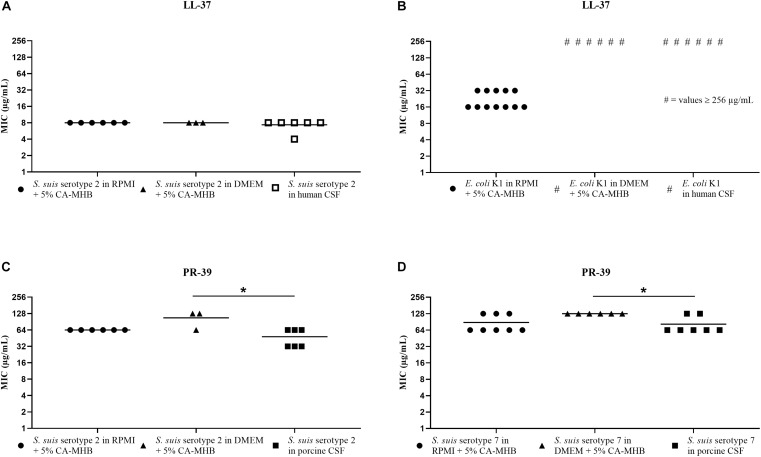
MIC testing in human and porcine CSF, RPMI + CA-MHB and DMEM + CA-MHB (data shown are values from three independent experiments). Test of *S. suis* serotype 2 against LL-37 **(A)** and PR-39 **(C)**, *E. coli* K1 against LL-37 **(B)** and *S. suis* serotype 7 against PR-39 **(D)**. Statistics: Kruskal–Wallis test with subsequent Dunn’s comparisons test ^∗^*p* < 0.0332.

The results of the MIC testing for human or porcine CSF are shown in Figure 3 and at the same time directly compared with MIC values obtained for RPMI + CA-MHB or DMEM + CA-MHB in the same assay. These results showed an MIC value of 8 μg/mL LL-37 in RPMI + CA-MHB and DMEM + CA-MHB, and 4 or 8 μg/mL in human CSF for the *S. suis* serotype 2 strain (Figure 3A). The *E. coli* K1 strain in RPMI + CA-MHB showed an MIC of 16 or 32 μg/mL. In DMEM + CA-MHB and human CSF, values of at least 256 μg/mL were determined (Figure 3B). Due to the fact that the MIC values were ≥256 μg/mL, no statistics were performed for this graph.

For PR-39, tested in porcine CSF with the *S. suis* serotypes 2 and 7 strains, we found MIC values that corresponded in principle to the values obtained in the RPMI + CA-MHB and DMEM + CA-MHB (Figures 3C,D). In detail, we found for *S. suis* serotype 2 (Figure 3C), MIC values of 64 μg/mL in RPMI + CA-MHB, 64 or 128 μg/mL in DMEM + CA-MHB and 32 or 64 μg/mL in porcine CSF. For *S. suis* serotype 7 (Figure 3D), MIC of 128 μg/mL in DMEM + CA-MHB and 64 or 128 μg/mL in RPMI + CA-MHB and porcine CSF were obtained.

In summary, for *S. suis* serotypes 2 and 7, the MIC values for PR-39 in porcine CSF were almost the same as in RPMI + CA-MHB and DMEM + CA-MHB (Figures 3C,D). In addition, for the combination of LL-37 and *S. suis* serotype 2, we found almost the same MIC in RPMI + CA-MHB, DMEM + CA-MHB and human CSF (Figure 3A). Therefore, we would recommend testing MICs of these AMPs against *S. suis* in RPMI or DMEM medium with 5% CA-MHB to get close to the MIC values of these peptides in CSF. For *E. coli* K1, we found out that this meningitis strain was not susceptible to LL-37 in CSF (Figure 3B).

It has already been shown that Gram-positive and Gram-negative bacteria react very differently to AMPs of one animal species (Langer et al., 2017). Due to this, it is not surprising that much higher MICs were found for the *E. coli* K1 strain than for *S. suis*. For *E. coli* K1 tested against LL-37 in human CSF, we found a much higher MIC than in CA-MHB or supplemented RPMI medium but the same MIC as shown in DMEM + CA-MHB (Figure 3B). This may be due to the polysialyl capsule, which may change in different media and increase the resistance of the bacterium to AMPs (Zelmer et al., 2008). Since *E. coli* K1 showed such high MIC values in CSF, MIC testing cannot be recommended in any medium because the physiological situation cannot be reflected.

The question is whether these detected MIC concentrations of AMPs can be reached in CSF in the infected host, and, thus, whether a direct antimicrobial effect against the pathogens is possible. Chen et al. (2004) demonstrated that the content of LL-37 in the lung greatly increased in parallel with the severity of lung disease. However, whether LL-37 or PR-39 can be transported through the blood CSF barrier or formed by local cells there and thereby reach high concentration gradients is still unclear. For other AMPs, transport through the barrier has already been shown (Stalmans et al., 2014). For CRAMP, an AMP of the mouse, it is known that it can be produced in endothelial cells of the blood brain barrier and in cells of the meninges (Bergman et al., 2006). The concentrations of AMPs that can be measured during meningitis in CSF are distinctly lower than the MICs we determined during our tests (Brandenburg et al., 2008; de Buhr et al., 2017). However, it is important to highlight that there may be a gradient of cathelicidins surrounding the activated leukocytes like van Harten et al. hypothesized (van Harten et al., 2018), so that inhibiting concentrations can be reached locally.

Besides the AMP gradient that can occur, the treatment with antimicrobials could have a synergistic effect with AMPs and thereby may improve the effect of antimicrobials, especially in the case of resistant bacteria (Lin et al., 2015). Interestingly, some antibiotics can increase the efficacy and binding capacity of AMPs to bacteria (Sakoulas et al., 2014). Not all bacteria react equally to chemotherapeutics in a particular medium. That is why Ersoy et al. (2017) recommend routine testing of the susceptibility of bacteria in the standard CA-MHB and additionally in a host-mimicking medium before giving an accurate statement about the antibacterial effect.

Finally, besides the direct antimicrobial effect on various bacteria, the cytotoxic activity also needs to be considered. For LL-37, the cytotoxic effect for cells has been shown with high concentrations at 13 μM LL-37 (Duplantier and van Hoek, 2013). The cytotoxic effects on brain cells are still unknown and require further investigations. However, the elevated hydrophobicity of AMPs is responsible for cell specificity toward bacteria and therefore low cytotoxicity for the host cell. A number of smaller synthetic peptidic analogs have similar antimicrobial activities compared with LL-37 but are less cytotoxic and thus may be useful tools for evaluating AMPs in the treatment of severe infections, e.g., chronic infected wounds (Duplantier and van Hoek, 2013). Also, for the screening of new synthetic AMP libraries, standardized and improved MIC testing with media closely mimicking physiological MIC values is needed.

In summary, in order to combat bacterial infections with alternatives to antimicrobials, e.g., cathelicidins, it is important to know whether these alternatives work in their natural environment. Their effect depends on peptide chain length, net charge and environmental conditions (Latendorf et al., 2019). Meningitis in piglets and human newborns is not easy to treat and without treatment usually leads to the death of the patient. The outcome of human neonatal meningitis caused by Gram-negative bacteria often leads to severe consequences (Basmaci et al., 2015). Therefore, it would be a great advantage if the effects of endogenous substances of the patients could be used for protective effects, either by external application or by indirect boosting of the host’s own expression. Efforts to use cathelicidins as a therapy are well advanced. Indeed, an LL-37 product from a Swedish company is already in Phase IIb of clinical testing for the therapy of chronic leg ulcers^1^ (Ekblom, 2013). Approaches to treating infections, inflammations and cancers of the oral mucosa are also well developed (Okumura, 2011).

On the way to discovering new effective AMPs, there is a need for susceptibility testing of AMPs in a medium that accurately mimics physiologically relevant conditions. However, it is difficult to find a suitable solution for all kinds of bacterial strains and AMP combinations. The suitability of different peptides is increasingly being tested (Jiao et al., 2017; Zhao et al., 2019). To establish a standardized method, we tried to find a medium that gives optimal results for Gram-positive and Gram-negative bacteria with similar effects of selected AMPs compared to human and porcine CSF. Based on our results, we can recommend RPMI + CA-MHB or DMEM + CA-MHBhttps://www.promorepharma.com/en/project-overview/

for susceptibility testing of *S. suis* against LL-37 and PR-39. *E. coli* K1 is not sensitive to LL-37 in CSF. Therefore, additional testing needs to be performed for putatively more active synthetic peptides.

## Data Availability Statement

All datasets generated for this study are included in the article/Supplementary Material.

## Ethics Statement

The use of pooled human CSF leftovers was reviewed and approved by the Ethics Committee of the University Medical Center Göttingen, Georg-August-University Göttingen. The animal study was reviewed and approved by the Lower Saxony State Office for Consumer Protection and Food Safety (Niedersächsisches Landesamt für Verbraucherschutz und Lebensmittelsicherheit).

## Author Contributions

MM, MK-B, NB, TG, JS, RN, CB, and SS designed the experiments. MM, LU, and MB performed the experiments. TG designed the peptides. MM, NB, and MK-B analyzed the data and drafted the manuscript. All authors proofread the manuscript.

## Conflict of Interest

The authors declare that the research was conducted in the absence of any commercial or financial relationships that could be construed as a potential conflict of interest.

## Footnotes

1 https://www.promorepharma.com/en/project-overview/
