# Supplementary material for: Comparing Cathelicidin Susceptibility of the Meningitis Pathogens Streptococcus suis and Escherichia coli in Culture Medium in Contrast to Porcine or Human Cerebrospinal Fluid
Source: Front Microbiol. 2020 Jan 14;10:2911. doi: 10.3389/fmicb.2019.02911 (PMC6971174; doi:10.3389/fmicb.2019.02911)
Supplement: Supplementary file 1 [file Data_Sheet_1.docx]

***Supplementary Material***

**Comparing cathelicidin susceptibility of the meningitis pathogens *Streptococcus suis* and *Escherichia coli* in culture medium in contrast to porcine or human cerebrospinal fluid**

Marita Meurer^1,2^, Nicole de Buhr^1,2^, Linn Meret Unger^1,2^, Marta C. Bonilla^1,2^, Jana Seele^3,4^, Roland Nau^3,4^, Christoph G. Baums^5^, Thomas Gutsmann^6^, Stefan Schwarz^7^, Maren von Köckritz-Blickwede^1,2^*

^1^Department of Physiological Chemistry, University of Veterinary Medicine Hannover, Foundation, Hannover, Germany; ^2^Research Center for Emerging Infections and Zoonoses (RIZ), University of Veterinary Medicine Hannover, Foundation, Hannover, Germany; ^3^Department of Neuropathology, University Medical Center Göttingen, Georg-August-University Göttingen, Göttingen, Germany; ^4^Department of Geriatrics, Evangelisches Krankenhaus Göttingen-Weende, Göttingen, Germany; ^5^Institute of Bacteriology and Mycology, Center for Infectious Diseases, Faculty of Veterinary Medicine, University Leipzig, Leipzig, Germany; ^6^Research group Biophysics, Research Center Borstel, Borstel, Germany; ^7^Institute of Microbiology and Epizootics, Centre for Infection Medicine, Department of Veterinary Medicine, Freie Universität Berlin, Berlin, Germany;

* E-Mail corresponding author:

maren.von.koeckritz-blickwede@tiho-hannover.de

**Supplementary Material and Methods:**

Synthesis of antimicrobial peptides

The peptides with amidated C-terminus were synthesized at the Research Center Borstel (Germany) in accordance with a standardized solid-phase Fmoc-synthesis protocol at 0.1 mmol scale (Andrä et al., 2009). Peptide synthesis was carried out in an automated experimental lapse on a peptide synthesizer 433A (Applied Biosystems, Carlsbad, CA, USA). The peptides were purified by HPLC and characterized by mass spectrometry. The purity was >95%. This purity for synthetic peptides means that a maximum of 5% of the peptides are shortened variants of the original peptide. These termination sequences were not further characterized. However, all shorter sequences investigated by us had a low activity. Thus, errors should be at a maximum of 5% reduced activity. Peptides were lyophilized and the weight used for the stock solutions (1 mg/mL) was determined with an error <5 %. Additionally, reference concentrations were measured photometrically showing variations of less than 20%. Both cathelicidins were dissolved in water and stored at -80 °C.

Growth curves

Microtiter plate wells (Greiner bio-one, PS, U-bottom 650101, without lid) were prepared with 50 µL CA-MHB, LB, porcine CSF, human CSF, RPMI or DMEM supplemented with 2.5 %, 5 %, 7.5 %, 10 %, 15 % and 20 % CA-MHB or 5 % laked horse blood. Subsequently, they were inoculated with 50 µL of a bacterial suspension in the same medium as had already been pipetted in the plate, with a concentration of 1x10^6^ CFU/mL, so the final concentration was 5x10^5^ CFU/mL. Wells were sealed with foil and incubated at 35 °C in the Tecan Plate Reader (Infinite 200 Pro) without CO_2_ or in the Tecan Plate Reader (Spark) with CO_2_ for 20 h. Every half hour, the OD_620nm_ was measured. Respective data are presented in Supplementary Figure 1.

Determination of bacterial growth

Five mL of each medium used for MIC testing was inoculated 1:100 with bacterial suspension prepared in 0.9 % NaCl solution, containing 1x10^8^ CFU/mL of each bacterial strain. After a six- and 22- hour incubation period at 35 °C with and without 5 % CO_2_ serial dilutions were plated on Columbia blood agar plates to determine the CFU/mL. Determining the CFU/mL in human or porcine CSF was performed after serial dilutions consisting of the 100µL volumes incubated for the growth curves in the Tecan Plate Reader (Spark). Respective data are presented in Supplementary Table 2.

LL-37 dot-blot analysis

To exclude the presence of LL-37 in the pooled human CSF samples, a dot-blot analysis was performed as previously described (de Buhr et al., 2017). To determine the detection limit, a standard row of 0.2, 1, 2 and 10µg LL-37 in 200 µL were blotted on a nitrocellulose membrane (Roth). Fifty µL of human CSF was diluted with 150 µL purified water to reach the working volume of 200 µL. The detection occurred by means of mouse‐anti‐LL‐37 (Hycult, 1:200, 1 % BSA in TBST) as first antibody and goat anti‐mouse HRP‐conjungated IgG (1:5000, 1 % BSA, TBST) as second antibody. Detection was done with SuperSignal West Pico Chemiluminescent Substrate (Thermo Scientific) as recommended by the manufacturer with ChemiDoc MP. Respective data are shown in Supplementary Figure 2.

PR-39 ELISA

To exclude the presence of PR‐39 in the porcine CSF samples, these were analyzed with PR-39 ELISA (Antibody Research Corporation, St Charles, MO, USA) according to the manufacturer's recommendation.

**Supplementary Figure 1:**

**Supplementary Figure 1**. A-D: Growth curves of *S. suis* serotype 2 (A, B), *E. coli* K12 (C, D) in RPMI or DMEM medium supplemented with different amounts of cation-adjusted Mueller-Hinton broth (CA-MHB). E-L: Comparison of growth curves in different media with and without CO_2_ for *S. suis* serotype 2 (E+F), *S. suis* serotype 7 (G+H), *E. coli* K1 (I+J) and *E. coli* K12 (K+L). M+N: Growth curves in human and porcine CSF. The bacterial growth at 35 °C was determined by measuring optical density at 620nm (OD_620nm_) every 30 min in the Tecan Plate reader (Infinite 200 Pro) without CO_2_ or in the Tecan Plate Reader (Spark) with CO_2_ for 20 h in a volume of 100 µL (n=1).

**Supplementary Figure 2**. LL-37 dot-blot analysis

|  | 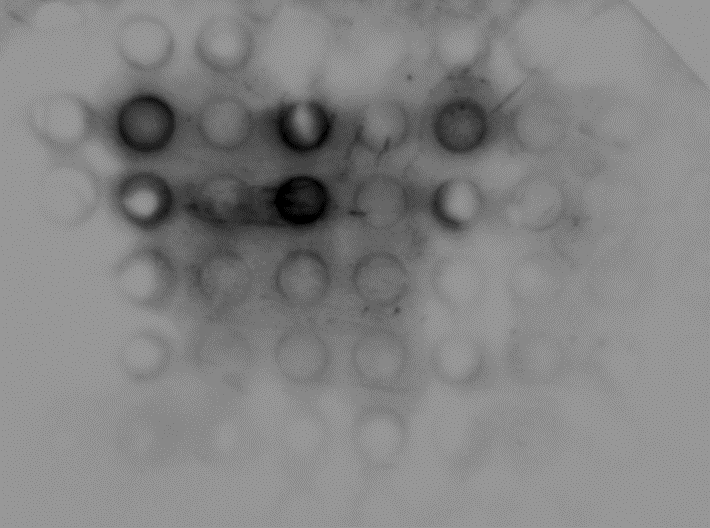 | 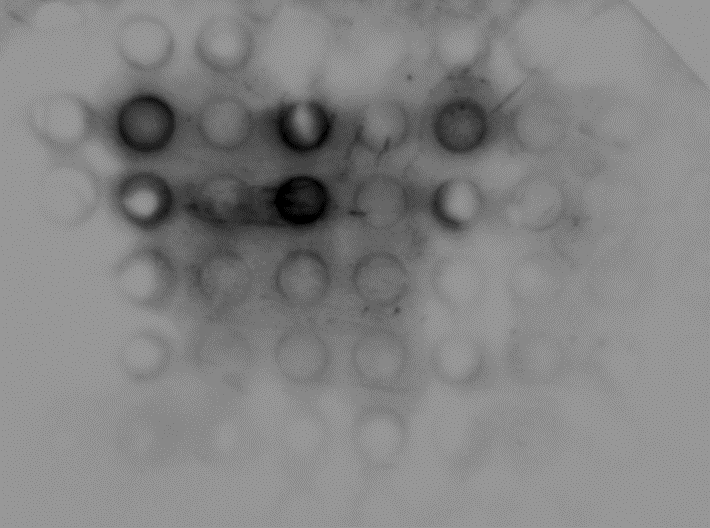 | 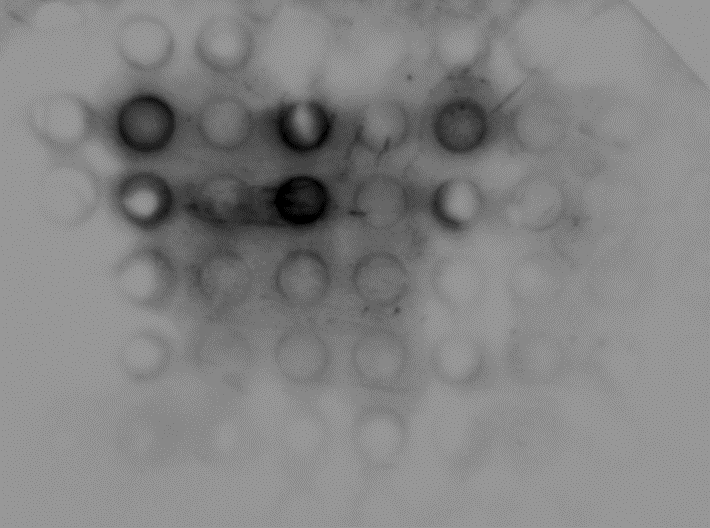 | 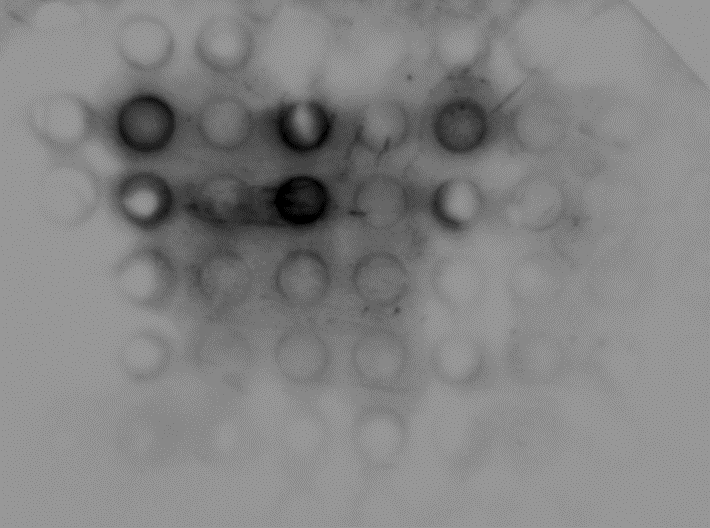 | 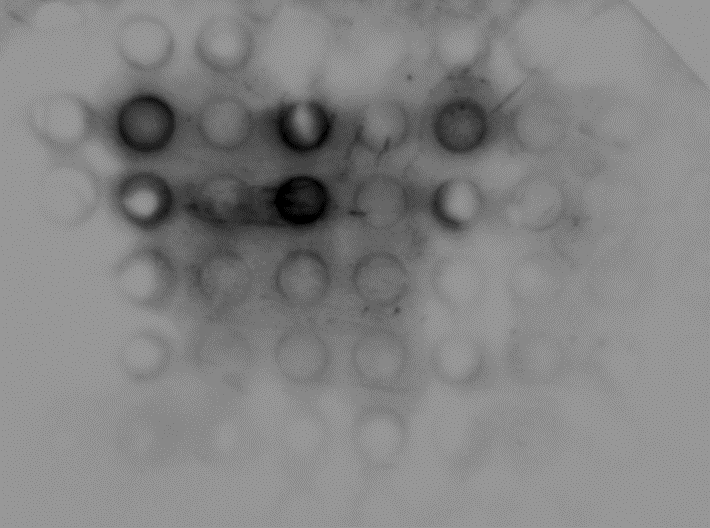 | 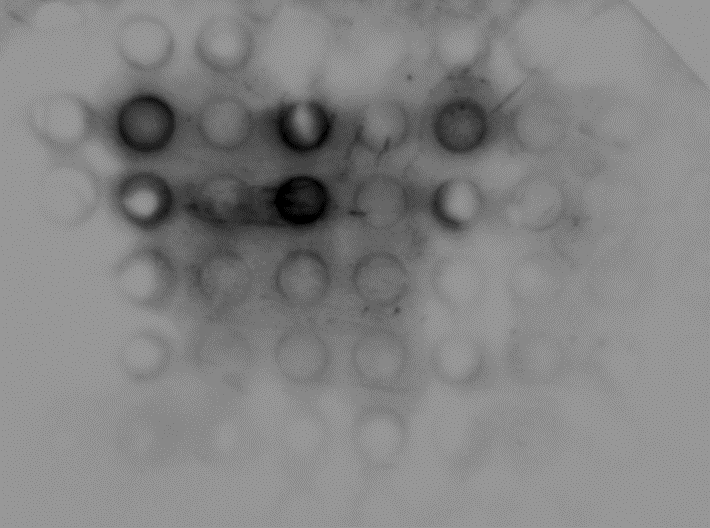 |
| --- | --- | --- | --- | --- | --- | --- |
| LL-37 | 10 µg | 2 µg | 1 µg | 0.2 µg | 0 µg | Human  CSF |

**Supplementary Figure 2**. Absence of relevant amount of LL-37 was proven by dot-blot analysis using mouse‐anti‐LL‐37 as first antibody and goat anti‐mouse HRP‐conjungated IgG as secondary antibody.

**Supplementary Table 1**. MIC values generated in accordance with CLSI recommendations by broth microdilution with two-fold dilution series and incubation at 35 °C with or without 5 % CO_2_ for 20 h. Data for all tested strain- medium-peptide combinations shown.

|  |  |  | MIC without CO_2_ | MIC with CO_2_ | MIC without CO_2_ | MIC with CO_2_ |
| --- | --- | --- | --- | --- | --- | --- |
| Strain | culture medium | replicate | LL-37  [µg/mL] | LL-37  [µg/mL] | PR-39  [µg/mL] | PR-39 [µg/mL] |
|  |  |  |  |  |  |  |
| ***S. suis*** | CA-MHB | 1 | 32 | 16 | 256 | 256 |
| **serotype 2** |  | 2 | 32 | 16 | 128 | 256 |
|  |  | 3 | 16 | 32 | 128 | 128 |
|  |  |  |  |  |  |  |
|  | RPMI | 1 | 4 | 4 | 32 | 32 |
|  | + 5 % CA-MHB | 2 | 4 | 8 | 32 | 64 |
|  |  | 3 | 4 | 4 | 64 | 64 |
|  |  | 4 |  | 8 |  | 64 |
|  |  | 5 |  | 8 |  | 64 |
|  |  | 6 |  | 8 |  | 64 |
|  |  | 7 |  | 8 |  | 64 |
|  |  | 8 |  | 8 |  | 64 |
|  |  | 9 |  | 8 |  | 64 |
|  |  |  |  |  |  |  |
|  | DMEM | 1 | 8 | 8 | 32 | 64 |
|  | + 5 % CA-MHB | 2 | 8 | 8 | 32 | 128 |
|  |  | 3 | 8 | 8 | 64 | 128 |
|  |  |  |  |  |  |  |
|  | RPMI | 1 | 64 | 64 | 32 | 64 |
|  | + 5 % blood | 2 | 64 | 64 | 64 | 64 |
|  |  | 3 | 128 | 128 | 64 | 64 |
|  |  |  |  |  |  |  |
|  | LB | 1 | 32 | 64 | 256 | 256 |
|  |  | 2 | 32 | 64 | 256 | 256 |
|  |  | 3 | 32 | 64 | 256 | 256 |
|  |  |  |  |  |  |  |
|  | human CSF | 1 |  | 8 |  |  |
|  |  | 2 |  | 8 |  |  |
|  |  | 3 |  | 8 |  |  |
|  |  | 4 |  | 8 |  |  |
|  |  | 5 |  | 8 |  |  |
|  |  | 6 |  | 4 |  |  |
|  |  |  |  |  |  |  |
|  | porcine CSF | 1 |  |  |  | 32 |
|  |  | 2 |  |  |  | 32 |
|  |  | 3 |  |  |  | 32 |
|  |  | 4 |  |  |  | 64 |
|  |  | 5 |  |  |  | 64 |
|  |  | 6 |  |  |  | 64 |

|  |  |  | MIC without CO_2_ | MIC with CO_2_ |
| --- | --- | --- | --- | --- |
| Strain | culture medium | replicate | PR-39 [µg/mL] | PR-39 [µg/mL] |
|  |  |  |  |  |
| ***S. suis*** | CA-MHB | 1 | ≥ 512 | ≥ 512 |
| **serotype 7** |  | 2 | ≥ 512 | ≥ 512 |
|  |  | 3 | 256 | ≥ 512 |
|  |  | 4 | 256 | ≥ 512 |
|  |  | 5 | 256 | 256 |
|  |  | 6 | 256 | 256 |
|  |  |  |  |  |
|  | RPMI | 1 | 64 | 64 |
|  | + 5 % CA-MHB | 2 | 64 | 64 |
|  |  | 3 | 64 | 128 |
|  |  | 4 | 64 | 64 |
|  |  | 5 | 64 | 64 |
|  |  | 6 | 64 | 64 |
|  |  | 7 |  | 128 |
|  |  | 8 |  | 128 |
|  |  |  |  |  |
|  | DMEM | 1 | 64 | 128 |
|  | + 5 % CA-MHB | 2 | 64 | 128 |
|  |  | 3 | 64 | 128 |
|  |  | 4 | 64 | 128 |
|  |  | 5 | 64 | 128 |
|  |  | 6 | 64 | 128 |
|  |  |  |  |  |
|  | RPMI | 1 | 128 | 128 |
|  | + 5 % blood | 2 | 128 | 128 |
|  |  | 3 | 128 | 256 |
|  |  | 4 | 128 | 256 |
|  |  | 5 | 256 | 128 |
|  |  | 6 | 128 | 256 |
|  |  |  |  |  |
|  | LB | 1 | ≥ 512 | ≥ 512 |
|  |  | 2 | ≥ 512 | ≥ 512 |
|  |  | 3 | 256 | 256 |
|  |  |  |  |  |
|  | porcine CSF | 1 |  | 128 |
|  |  | 2 |  | 128 |
|  |  | 3 |  | 64 |
|  |  | 4 |  | 64 |
|  |  | 5 |  | 64 |
|  |  | 6 |  | 64 |
|  |  | 7 |  | 64 |

|  |  |  | MIC without CO_2_ | MIC with CO_2_ |
| --- | --- | --- | --- | --- |
| Strain | culture medium | replicate | LL-37 [µg/mL] | LL-37 [µg/mL] |
|  |  |  |  |  |
| ***E. coli* K1** | CA-MHB | 1 | 128 | 128 |
|  |  | 2 | 128 | 128 |
|  |  | 3 | 64 | 128 |
|  |  | 4 | 64 | 128 |
|  |  | 5 | 64 | 64 |
|  |  | 6 | 64 | 64 |
|  |  | 7 | 64 | 64 |
|  |  | 8 | 64 | 64 |
|  |  | 9 | 64 | 64 |
|  |  | 10 |  | 64 |
|  |  |  |  |  |
|  |  |  |  |  |
|  | RPMI | 1 | 32 | 32 |
|  | + 5 % CA-MHB | 2 | 32 | 32 |
|  |  | 3 | 16 | 32 |
|  |  | 4 | 16 | 32 |
|  |  | 5 | 16 | 32 |
|  |  | 6 | 16 | 16 |
|  |  | 7 | 16 | 16 |
|  |  | 8 | 16 | 16 |
|  |  | 9 | 16 | 16 |
|  |  | 10 |  | 16 |
|  |  | 11 |  | 16 |
|  |  | 12 |  | 16 |
|  |  |  |  |  |
|  | DMEM | 1 | 256 | ≥ 512 |
|  | + 5 % CA-MHB | 2 | 256 | ≥ 512 |
|  |  | 3 | ≥ 512 | ≥ 512 |
|  |  | 4 | ≥ 512 | ≥ 512 |
|  |  | 5 | ≥ 512 | ≥ 512 |
|  |  |  |  |  |
|  | LB | 1 | 128 | 128 |
|  |  | 2 | 128 | 128 |
|  |  | 3 | 128 | 128 |
|  |  | 4 | 128 | 128 |
|  |  | 5 | 128 | 256 |
|  |  | 6 | 128 | 128 |
|  |  |  |  |  |
|  | human CSF | 1 |  | ≥ 256 |
|  |  | 2 |  | ≥ 256 |
|  |  | 3 |  | ≥ 256 |
|  |  | 4 |  | ≥ 256 |
|  |  | 5 |  | ≥ 256 |
|  |  | 6 |  | ≥ 256 |
|  |  |  |  |  |
|  | porcine CSF | 1 |  | ≥ 256 |

|  |  |  | MIC without CO_2_ | MIC with CO_2_ | MIC without CO_2_ | MIC with CO_2_ |
| --- | --- | --- | --- | --- | --- | --- |
| Strain | culture medium | replicate | LL-37  [µg/mL] | LL-37  [µg/mL] | PR-39  [µg/mL] | PR-39  [µg/mL] |
|  |  |  |  |  |  |  |
| ***E. coli* K12** | CA-MHB | 1 | 32 | 32 | 4 | 8 |
|  |  | 2 | 32 | 32 | 4 | 8 |
|  |  | 3 | 32 | 32 | 8 | 8 |
|  |  |  |  |  |  |  |
|  | RPMI | 1 | 4 | 16 | 4 | 4 |
|  | + 5 % CA-MHB | 2 | 8 | 16 | 4 | 4 |
|  |  | 3 | 16 | 16 | 4 | 4 |
|  |  |  |  |  |  |  |
|  | DMEM | 1 | 64 | 64 | 4 | 8 |
|  | + 5 % CA-MHB | 2 | 64 | 64 | 8 | 4 |
|  |  | 3 | 64 | 64 | 8 | 8 |
|  |  |  |  |  |  |  |
|  | LB | 1 | 128 | 128 | 32 | 32 |
|  |  | 2 | 128 | 128 | 32 | 32 |
|  |  | 3 | 128 | 128 | 32 | 32 |

|  |  |  | MIC without CO_2_ | MIC with CO_2_ |
| --- | --- | --- | --- | --- |
| Strain | culture medium | replicate | LL-37  [µg/mL] | LL-37  [µg/mL] |
|  |  |  |  |  |
| ***S. aureus*** | MHB | 1 | 8 | 8 |
| **Newman dlt** |  | 2 | 8 | 8 |
|  |  | 3 | 8 | 8 |
|  |  | 4 | 8 | 8 |
|  |  | 5 | 8 | 8 |
|  |  | 6 | 8 | 8 |
|  |  |  |  |  |
|  | CA-MHB | 1 | 16 | 16 |
|  |  | 2 | 8 | 16 |
|  |  | 3 | 8 | 8 |
|  |  | 4 | 8 | 8 |
|  |  |  |  |  |
|  | RPMI | 1 | 4 | 8 |
|  | + 5 % CA-MHB | 2 | 4 | 8 |
|  |  | 3 | 4 | 8 |
|  |  | 4 | 4 | 8 |
|  |  |  |  |  |
|  | DMEM | 1 | 16 | 16 |
|  | + 5 % CA-MHB | 2 | 16 | 16 |
|  |  | 3 | 4 | 8 |
|  |  | 4 | 4 | 8 |

**Supplementary Table 2.** Strains were grown in 5ml of different media without (A) and with (B) CO_2_ incubation at 35 °C. After a six and 22 hours incubation period, the OD _620nm_ and CFU/mL were determined. The values for human and porcine CSF were generated in 100 µl (n =1).

**Supplementary Table 2A**. OD _620nm_ and CFU/mL after a 6 and 22 h incubation period at 35 °C without CO_2_.

|  |  | OD_620nm_ | CFU/mL | OD_620nm_ | CFU/mL |
| --- | --- | --- | --- | --- | --- |
| Strain | culture medium | 6h | 6h | 22h | 22h |
| ***S. suis*** | CA-MHB | 0.350 | 5.00E+08 | 0.367 | 1.50E+08 |
| **serotype 2** | RPMI + 5 % CA-MHB | 0.230 | 4.60E+08 | 0.362 | 2.88E+08 |
|  | DMEM + 5 % CA-MHB | 0.223 | 4.13E+08 | 0.369 | 2.53E+08 |
|  | LB | 0.298 | 3.23E+08 | 0.194 | 9.50E+05 |
|  | RPMI + 5 % lysed horse blood | 0.805 | 6.38E+08 | 1.267 | 1.00E+09 |
|  |  |  |  |  |  |
| ***S. suis*** | CA-MHB | 0.479 | 2.55E+08 | 0.463 | 4.05E+04 |
| **serotype 7** | RPMI + 5 % CA-MHB | 0.747 | 9.28E+08 | 0.256 | 2.00E+08 |
|  | DMEM + 5 % CA-MHB | 0.654 | 6.38E+08 | 1.139 | 0.00E+00 |
|  | LB | 0.165 | 1.45E+08 | 0.204 | 5.13E+06 |
|  | RPMI + 5 % lysed horse blood | 0.929 | 8.75E+08 | 1.401 | 1.10E+09 |
|  |  |  |  |  |  |
| ***E. coli* K1** | CA-MHB | 1.024 | 7.33E+08 | 1.405 | 1.10E+08 |
|  | RPMI + 5 % CA-MHB | 1.067 | 6.28E+08 | 1.242 | 1.10E+08 |
|  | DMEM + 5 % CA-MHB | 0.999 | 6.18E+08 | 1.33 | 9.00E+07 |
|  | LB | 0.804 | 5.20E+08 | 1.171 | 7.50E+07 |
|  |  |  |  |  |  |
| ***E. coli* K12** | CA-MHB | 0.820 | 4.40E+08 | 1.262 | 1.25E+08 |
|  | RPMI + 5 % CA-MHB | 0.943 | 5.95E+08 | 1.046 | 1.05E+08 |
|  | DMEM + 5 % CA-MHB | 0.882 | 1.65E+08 | 1.135 | 2.00E+07 |
|  | LB | 0.582 | 3.88E+08 | 1.477 | 1.65E+08 |

**Supplementary Table 2B**. OD _620nm_ and CFU/mL after 6 and 22 h incubation at 35 °C with CO_2_

|  | | | | | |
| --- | --- | --- | --- | --- | --- |
|  |  | OD_620nm_ | CFU/mL | OD_620nm_ | CFU/mL |
| Strain | culture medium | 6h | 6h | 22h | 22h |
| ***S. suis*** | CA-MHB | 0.356 | 3.90E+08 | 0.304 | 1.40E+07 |
| **serotype 2** | RPMI + 5 % CA-MHB | 0.192 | 3.05E+08 | 0.345 | 3.65E+08 |
|  | DMEM + 5 % CA-MHB | 0.193 | 4.35E+08 | 0.368 | 2.93E+08 |
|  | LB | 0.280 | 2.35E+08 | 0.202 | 4.35E+05 |
|  | RPMI + 5 % lysed horse blood | 0.607 | 6.13E+08 | 1.283 | 1.00E+09 |
|  | human CSF |  |  | 0.153 | 3.93E+07 |
|  | porcine CSF |  |  | 0.151 | 7.53E+07 |
|  |  |  |  |  |  |
| ***S. suis*** | CA-MHB | 0.437 | 4.58E+08 | 0.429 | 1.65E+04 |
| **serotype 7** | RPMI + 5 % CA-MHB | 0.437 | 8.10E+08 | 0.421 | 2.15E+07 |
|  | DMEM + 5 % CA-MHB | 0.388 | 5.15E+08 | 1.116 | 5.50E+03 |
|  | LB | 0.084 | 6.15E+07 | 0.235 | 2.05E+06 |
|  | RPMI + 5 % lysed horse blood | 0.704 | 9.63E+08 | 1.116 | 9.50E+08 |
|  | porcine CSF |  |  | 0.193 | 1.90E+08 |
|  |  |  |  |  |  |
| ***E. coli* K1** | CA-MHB | 0.895 | 3.23E+08 | 1.47 | 8.50E+08 |
|  | RPMI + 5 % CA-MHB | 0.890 | 3.25E+08 | 1.373 | 9.00E+08 |
|  | DMEM + 5 % CA-MHB | 0.646 | 2.33E+08 | 1.377 | 8.00E+08 |
|  | LB | 0.708 | 4.13E+08 | 1.160 | 7.50E+08 |
|  | human CSF |  |  | 0.266 | 4.40E+08 |
|  | porcine CSF |  |  | 0.319 | 6.85E+08 |
|  |  |  |  |  |  |
| ***E. coli* K12** | CA-MHB | 0.667 | 3.18E+08 | 1.117 | 1.00E+09 |
|  | RPMI + 5 % CA-MHB | 0.767 | 3.00E+08 | 1.205 | 6.50E+08 |
|  | DMEM + 5 % CA-MHB | 0.645 | 5.00E+07 | 1.113 | 5.00E+07 |
|  | LB | 0.514 | 3.08E+08 | 1.495 | 1.75E+09 |

**References:**

Andrä, J., Hammer, M. U., Grötzinger, J., Jakovkin, I., Lindner, B., Vollmer, E., et al. (2009). Significance of the cyclic structure and of arginine residues for the antibacterial activity of arenicin-1 and its interaction with phospholipid and lipopolysaccharide model membranes. *Biol. Chem.* 390, 337–349. doi:10.1515/BC.2009.039.

de Buhr, N., Reuner, F., Neumann, A., Stump-Guthier, C., Tenenbaum, T., Schroten, H., et al. (2017). Neutrophil extracellular trap formation in the Streptococcus suis-infected cerebrospinal fluid compartment. *Cell. Microbiol.* 19. doi:10.1111/cmi.12649.
